# Homocysteine induces ferroptosis in cardiomyocytes by disrupting β-catenin/GPX4 pathway

**DOI:** 10.1371/journal.pone.0329792

**Published:** 2025-08-06

**Authors:** Yanping Lei, Rui Liu, Lewu Xu, Yue Zhao

**Affiliations:** 1 Institute of Cardiovascular Disease, Key Laboratory for Arteriosclerology of Hunan Province, Hunan International Scientific and Technological Cooperation Base of Arteriosclerotic Disease, Hengyang Medical College, University of South China, Hengyang, Hunan, China; 2 The First Affiliated Hospital, Department of Cardiology, Hengyang Medical School, University of South China, Hengyang, Hunan, China; University of Pennsylvania, UNITED STATES OF AMERICA

## Abstract

**Background:**

Homocysteine can cause damage to cardiomyocytes, but the exact mechanism underlying that injury is unknown. And, ferroptosis contributes to both the initiation and progression of cardiac diseases. This study aims to focus on homocysteine to investigate the involvement of β-catenin/GPX4 signaling in ferroptosis of cardiomyocytes.

**Methods:**

In this study, C57BL/6 mice were utilized to establish an experimental model. Hyperhomocysteinemia was induced in the animal model by administering homocysteine at a concentration of 1.8 g/L in the drinking water. Model mice received the treatment of deferoxamine (DFO) and ferrostatin-1 (Fer-1) as therapeutic interventions. Western blot was utilized to detect β-catenin, FTH1, and GPX4. Lipid ROS, Fe^2+^, and GSH were detected by biochemical assays. In addition, β-catenin and GPX4 expression were assessed by immunostaining techniques. Cell viability was assessed using CCK-8 assay, and mitochondrial damage was examined by transmission electron microscopy. ChIP combining dual luciferase reporter gene assay was performed to analyze the interaction between β-catenin protein with the promoter of GPX4 gene.

**Results:**

Homocysteine inhibited β-catenin activity and GPX4 expression, and promoted cardiomyocytes ferroptosis *in vitro* and *in vivo*. Overexpression of β-catenin promoted the expression of GPX4 and subsequently inhibited homocysteine-induced ferroptosis in cardiomyocytes. Further, results from the ChIP assay and dual-luciferase reporter assay indicated that GPX4 acted as a target gene of β-catenin.

**Conclusion:**

Homocysteine induces ferroptosis in cardiomyocytes by disrupting β-catenin activity, subsequently downregulating its target gene, GPX4.

## Introduction

Homocysteine (Hcy) serves as an intermediate metabolite in the cycle of cysteine and methionine, primarily existing in the form of protein binding [1]. Hcy-induced damage affects various biochemical processes, including protein synthesis, DNA synthesis, and methylation, leading to structural damage in molecules such as proteins and DNA [2]. Consequently, these damages contribute to the onset of various diseases such as chronic kidney disease, osteoporosis, and cardiovascular disease [1–3]. Epidemiological studies identify hyperhomocysteinemia as a major risk for multiple cardiovascular issues such as cardiac hypertrophy, coronary artery disease, and arrhythmias [4–6]. However, the precise mechanism governing homocysteine-induced injury remain unclear. Recent studies suggest an association between ferroptosis and homocysteine-induced cellular damage [7]. Ferroptosis represents a unique form of regulated cell death, different from both necrosis and apoptosis. Ferroptosis is driven by an elevation of intracellular ferrous ions (Fe^2+^), characterized by inhibition of glutathione peroxidase 4 (GPX4), a decline in glutathione (GSH), and the generation of lipid reactive oxygen species (ROS). GPX4, an inhibitor for ferroptosis, converts lipid peroxides into harmless lipid alcohols, thus attenuating the toxicity of lipid peroxides [8]. Ferroptosis is marked by defects in mitochondrial membrane integrity, such as disruption of the mitochondrial membrane, mitochondrial shrinkage, and mitochondrial cristae reduction. Classical inhibitors of ferroptosis include deferoxamine (DFO) and ferrostatin-1 (Fer-1) [9]. In our preliminary experiments, we found that homocysteine induced the downregulation of β-catenin and GPX4 in cardiomyocytes. As a ligand of conserved β-catenin signaling, Wnt binds to membrane receptors to prevent the β-catenin degradation, resulting in its accumulation in cytoplasm and subsequent activation of target gene transcription [10]. As a highly conserved signaling molecule, activated β-catenin signaling promotes cells proliferation and repair against stresses such as inflammation and injury [11–13]. β-catenin signaling plays key role in the developmental processes of the heart [14]. In the heart, β-catenin signaling is generally sustained at a low basal level, which is still vital for sustaining heart function. Based on existing literature and our preliminary findings, this study aims to explore whether homocysteine induces cardiomyocyte ferroptosis and seeks to elucidate the roles of both β-catenin and GPX4 in homocysteine-driven cardiomyocyte ferroptosis.

## Materials and methods

### Animal models

SPF (Specific pathogen free) male C57BL/6J mice (8 weeks old, weighing 15 ~ 20 g) were acquired from Hunan Silaike Jingda Laboratory Animal Co. Ltd (Changsha, China). Laboratory mice were assigned into 6 groups randomly (n = 4): the control group, the model group (Homocysteine was introduced into the drinking water at a level of 1.8 g/L), DFO treatment group (Model mice received subcutaneous injections of DFO at a dosage of 100 mg/kg/day), Fer-1 treatment group (Model mice received intraperitoneal administration of Fer-1 at a dosage of 2 mg/kg/day). After the 4th week, mice were euthanized by subcutaneously injecting pentobarbital sodium (30 mg/kg). All animal experiments had been approved by the Animal Ethics Committee at the University of South China, located in Hengyang, China (Ethics approval number: USC2023XS087).

### Cell culture

Primary cardiomyocytes of neonatal mice were collected following the method described previously [15]. The proportion of primary cardiomyocytes in the isolated cell pool was more than 95%, as evidenced by immunostaining for α-actin. Cardiomyocytes were cultivated with DMEM/F12 with 10% bovine serum. Cells underwent serum-starvation for 12 hours prior to a variety of treatments. Cardiomyocytes were treated with homocysteine (1 mmol/L). DFO (500 μmol/L) and Fer-1 (10 μmol/L) were administered as the treatments, and the incubation time was 12 hours. Then, cells were collected for immunofluorescence staining, electron microscope examination, and western blot, respectively.

### Cell transfection with plasmids or siRNAs

Cardiomyocytes were cultivated on 6-well plates. Cells were prepared for transfection after the cell confluence reaching approximately 80%. Cardiomyocytes were transfected using lipofectamine with pcDNA3.1-β-catenin (2.5 μg) or pcDNA3.1-GPX4 (2.5 μg). A total of 75 pmol of small interfering RNA targeting β-catenin (β-catenin-siRNA) was mixed with liposome at room temperature for 20 minutes to generate liposome complex. The incubation of cells and liposome complex lasted for 6 hours. Lipofectamine 3000 (Invitrogen, USA) and Opti-MEM (Gibco, USA) were utilized for the transfection of plasmids and siRNAs. The siRNA sequences were as follows: the β-catenin-siRNA sequence 5’-GCCUCUGAUAAAGGCAACUTT-3’, and the β-catenin scramble sequence 5’-CAGUACUUUUGUGUAGUACAA-3’.

### Western blot

Western blotting was performed for protein quantification following standard procedures. The primary antibodies used were listed as follows: β-catenin (610154; BD Biosciences, CA), active β-catenin (#19807, Cell Signaling Technology, MA), GPX4 (Ab125066, Abcam), FTH1 (Ab183781, Abcam) and β-actin (AA128, Beyotime Biotechnology). The levels of protein expression were normalized using β-actin.

### Immunohistochemical staining

The preparation of paraffin sections was conducted following the protocol as previously described [16]. Heart sections were immunostained for target proteins with primary antibodies for β-catenin (610154; BD Biosciences, CA) and GPX4 (Ab125066; Abcam). All images were acquired on a bright-field microscope (Nikon).

### Immunofluorescent staining

The primary cardiomyocytes from neonatal mice were seeded on the coverslips. Cardiomyocytes were fixed in 4% methanal buffer for 20 minutes, followed by a 30-minute blocking with 10% bovine serum. The slides were incubated with the specified primary antibodies, and Cy3-conjugated secondary antibodies (A0516, beyotime biotechnology) in turn. Then, the nuclei was stained with DAPI for visualization. All micrographs were acquired on a Leica fluorescence microscope.

### GSH (Glutathione) assay

GSH levels were tested with a kit (#A006-2, Jiancheng, Nanjing) following manufacturer’s instruction. The abundance of GSH was detected using a spectrophotometer at 420 nm. GSH levels were standardized to the total protein levels.

### Labile iron assay

Labile iron (Fe^2+^) concentrations in cardiomyocytes and cardiac tissue were assessed with a kit (Abcam, #ab83366), following the instructions of manufacturer.

### Lipid ROS assay

Lipid ROS levels were tested by BODIPY581/591 C11, a fluorescent probe (D3861, Invitrogen, USA). Cardiomyocytes were transfected with pcDNA3.1-β-catenin, then stimulated with homocysteine, and incubated with BODIPY581/591 C11 at a level of 10 μM for 30 minutes in darkness. Cells were visualized on the ﬂuorescence microscope (Leica, Wetzlar, Germany). Malondialdehyde (MDA) was quantified via thiobarbituric acid to examine lipid ROS and MDA assay kit was purchased from Beyotime (Nanjing, China).

### Transmission electron microscopy (TEM) detection

TEM (Transmission electron microscopy) was employed to observe mitochondrial injury and mitophagy in cells and tissues. Small cubic pieces of myocardium (1–2 mm^3^) and cardiomyocytes were sequentially fixed with glutaraldehyde (2.5%) and osmic acid (1%). Subsequently, the samples were embedded, sectioned and double-stained with uranium acetate (3%) and lead citrate. Ultimately, the mitochondria were examined using TEM (Hitachi).

### Chromatin immunoprecipitation (ChIP)

The pcDNA3.1-β-catenin was utilized to transfected H9c2 cells. After 48 hours, cells underwent being fixed in 4% formaldehyde buffer to facilitate protein-DNA crosslinking. ChIP was carried out by utilizing the SimpleChIP Plus Kit (Cat. 9005, Cell Signaling). The antibody against β-catenin (ab32572, abcam), RNAP Ⅱ (RNA polymerase Ⅱ), and rabbit IgG were added for incubation overnight at 4 °C. Subsequently, protein A-agarose was added and incubated for 1 hour. After washing, the purified DNA was used for PCR amplification. The primer pair designed for the GPX4 gene promoter included: forward primer 5’- AAGCCAGGTTTCCTTGTGTG-3’ and reverse primer 5’- ATGCCTGTGACTGTACATGC-3’.

### RT-PCR

To synthesize the first strand cDNA, 2 µg of RNA were added as templates to a 20 µl reverse transcription reaction system (RK20433, ABclonal). Reverse transcription-polymerase chain reaction (RT-PCR) was carried out on an Applied Biosystems (2720, America). RT-PCR was performed for mRNA quantification following standard procedures [17]. The primer pair designed for GPX4 PCR was listed as follows: sense primer 5’-CAACCAGTTCGGGAGGCAGGAG-3’ and antisense primer 5’- TGGGCTGGACTTTCATCCATTTC-3’.

### Dual luciferase reporter gene assay

Reporter gene plasmids (pRL-SV40-C and pGL6-TA) and Dual luciferase reporter gene assay kit were purchased from Beyotime Biotechnology. Both the pRL-SV40-C and pGL6-TA-GPX4 promoter were co-transfected into HEK293T cells along with pcDNA3.1-β-catenin plasmid. After 48 hours of transfection, the cells were subjected to lysis and centrifugation to obtain the supernatant. Samples were loaded into a 96-well microplate, and fluorescence intensity was determined on a luminometer (Bio Tek, Synergy, USA). Luciferase activity was evaluated by the intensity of firefly fluorescence, which was standardized by the intensity of renilla fluorescence.

### CCK-8 detection

Cell viability was examined with CCK-8 kit (CK04, DOJINDO) following manufacture’s instruction.

### Statistical analyses

All data were expressed as mean ± SEM in our study. Statistical analysis was perform with GraphPad Prism (La Jolla, CA). The comparison among groups was implemented with one-way ANOVA. And statistical analyses between two groups was carried out by Student-Newman-Kuels test. P < 0.05 was a signiﬁcant difference of statistic.

## Results

### Homocysteine induced ferroptosis and downregulated the levels of β-catenin and GPX4 in hearts

**Fig 1:**
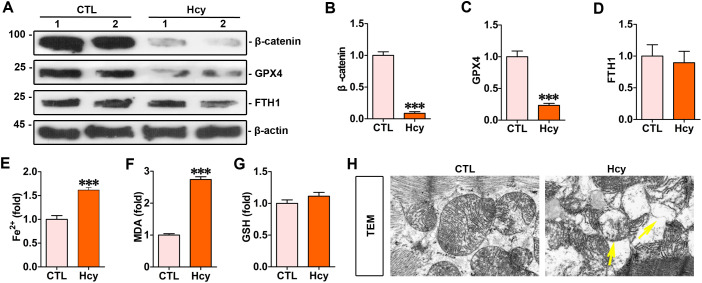
Homocysteine induced ferroptosis and downregulated the levels of β-catenin and GPX4 in hearts. **(A)** Western blot analyses showed the expression of proteins including β-catenin, GPX4 and FTH1 in the heart of mice subjected to hyperhomocysteinemia for 4 weeks. **(B-D)** Quantitative data on β-catenin, GPX4 and FTH1 proteins in indicated groups. Relative levels of proteins were presented as fold induction over the controls. ***P < 0.001 (n = 4). **(E-G)** Quantitative determination of Fe^2+^, lipid ROS and GSH on the colorimetric microplate reader. ***P < 0.001 (n = 4). **(H)** Representative transmission electron microscope images showed mitochondrial damage in the heart of mice subjected to hyperhomocysteinemia for 4 weeks. The yellow arrows indicated injured mitochondria. Scale bar, 300 nm.

The hyperhomocysteinemia mouse model was established by providing drinking water with a high level of homocysteine. The myocardial β-catenin and GPX4 in mice with hyperhomocysteinemia were markedly lower than the control group, whereas the expression of FTH1 remained unchanged (Figs 1A-1D). Biochemical assays were used to examine the concentrations of Fe^2+^, Lipid ROS, and GSH in the myocardium (Figs 1E-1G). The levels of myocardial Fe^2+^ and lipid ROS were considerably higher in mice with hyperhomocysteinemia compared with the control mice (Figs 1E and 1F). Moreover, transmission electron microscopy showed that homocysteine induced characteristic mitochondrial injury associated with ferroptosis in cardiomyocytes, as indicated by yellow arrows (Fig 1H).

### DFO and Fer-1 alleviated ferroptosis induced by homocysteine without affecting β-catenin and GPX4 expression in mouse hearts

**Fig 2:**
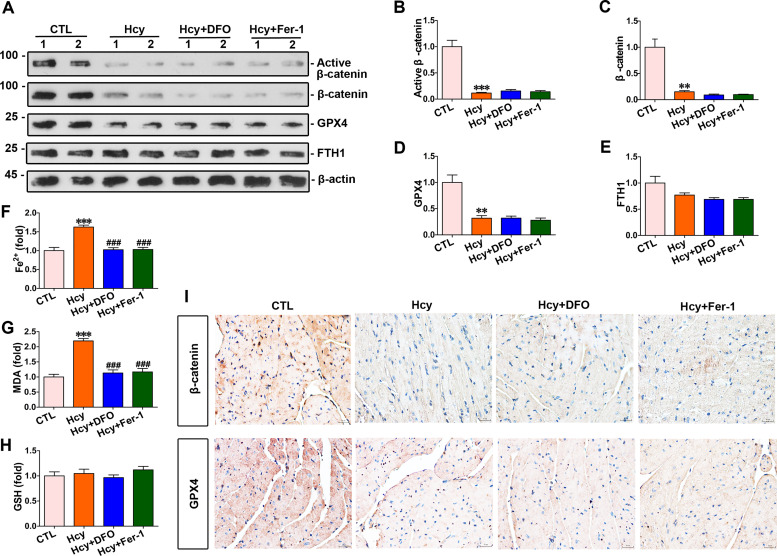
DFO and Fer-1 respectively inhibited homocysteine-induced ferroptosis in the heart of mouse. **(A)** Western blot analyses showed the expression level of β-catenin, active β-catenin, GPX4 and FTH1 in the hearts of mice subjected to hyperhomocysteinemia for 4 weeks. (B-E) quantitative data for protein levels in Fig 2A were presented. ***P < 0.001, **P < 0.01, *P < 0.05 *vs* the controls (n = 4). **(F-H)** Quantitative determination of Fe^2+^, lipid ROS and GSH in indicated groups on the colorimetric microplate reader. ***P < 0.001, **P < 0.01, *P < 0.05 *vs* the controls; ^###^*P* < 0.001, ^##^*P* < 0.01, ^#^*P* < 0.05 *vs* hyperhomocysteinemia mice (n = 4). **(I)** Representative micrographs showed staining for β-catenin and GPX4 proteins in the hearts of mice at the end of 4th week of hyperhomocysteinemia model. Upper panel, immunostaining for β-catenin in the hearts of mice as indicated; Bottom panel, immunostaining for GPX4 in given groups as indicated. Scale bar, 20 μm.

The DFO and Fer-1 were administered to mice as treatment measures. Western blot indicated that homocysteine downregulated of total β-catenin, active β-catenin, and GPX4; however, the levels of these proteins in the myocardium of mice treated with DFO or Fer-1 were comparable to those in homocysteine-stimulated mice; there was no significant difference in FTH1 expression among all groups (Figs 2A–2E). Myocardial concentrations of Fe^2+^ and lipid ROS were significantly reduced in DFO or Fer-1 treatment mice compared to homocysteine-stimulated mice; GSH levels remained unchanged among all the groups (Figs 2F–2H). Further immunohistochemical staining revealed that both β-catenin and GPX4 expressions were markedly reduced in the hearts of hyperhomocysteinemic mice compared to the control group; neither DFO nor Fer-1 treatments had any effect on β-catenin and GPX4 expression (Fig 2I).

### Both DFO and Fer-1 inhibited cardiomyocyte ferroptosis induced by homocysteine, without affecting β-catenin and GPX4 expression

**Fig 3:**
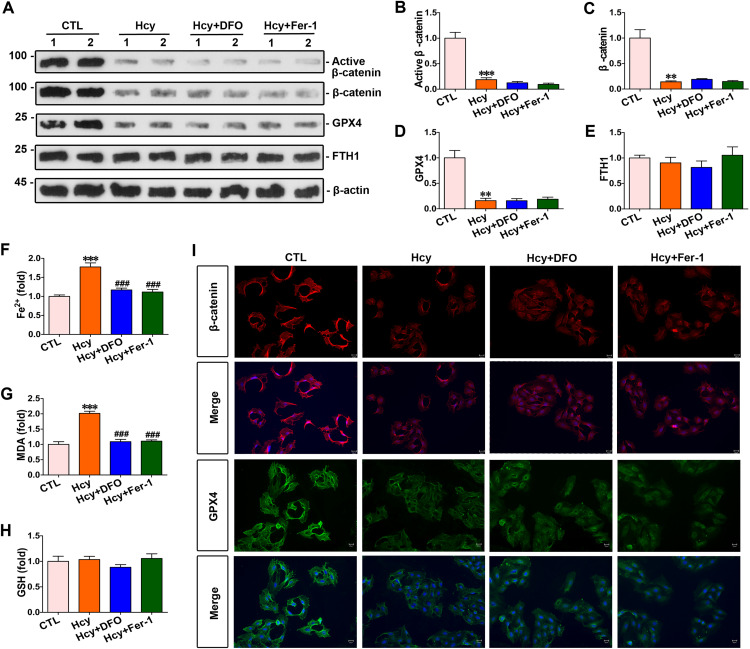
DFO and Fer-1 respectively inhibited homocysteine-induced ferroptosis in cardiomyocytes. **(A)** Western blots showed protein levels of β-catenin, active β-catenin, GPX4 and FTH1 in cardiomyocytes of each group as indicated. **(B-E)** Quantitative data on the abundance of specific proteins of Fig 3A were presented in indicated groups. ***P < 0.001, **P < 0.01, *P < 0.05 *vs* the controls (n = 4). **(F-H)** Quantitative determination of Fe^2+^, lipid ROS and GSH in indicated groups on the colorimetric microplate reader. ***P < 0.001, **P < 0.01, *P < 0.05 *vs* the controls; ^###^*P* < 0.001, ^##^*P* < 0.01, ^#^*P* < 0.05 *vs* homocysteine stimulation alone (n = 4). **(I)** Representative micrographs showed staining for β-catenin and GPX4 proteins in cardiomyocytes. Upper panel, immunofluorescent staining for β-catenin in given groups; Bottom panel, immunostaining for GPX4 in cardiomyocytes as indicated groups. Scale bar, 25 μm.

Primary mouse cardiomyocytes were stimulated with homocysteine. and DFO and Fer-1, were added as treatments, respectively. Western blot results revealed that homocysteine inhibited the expression of total β-catenin, active β-catenin and GPX4, while it had no impact on the expression of FTH1; and the levels of total β-catenin, active β-catenin, GPX4, and FTH1 in cardiomyocytes treated with DFO or Fer-1 were comparable to those in homocysteine-stimulated myocytes (Figs 3A–3E). Homocysteine markedly increased intracellular Fe²⁺ and lipid ROS levels; compared with homocysteine-stimulated cardiomyocytes, both DFO and Fer-1 treatments significantly decreased intracellular Fe^2+^ and lipid ROS levels (Figs 3F and 3G). Further, immunofluorescence showed that β-catenin and GPX4 expression levels in cardiomyocytes were significantly decreased after homocysteine stimulation compared with the control group, while both DFO and Fer-1 treatments had no impact on β-catenin and GPX4 levels (Fig 3I).

### Increased β-catenin expression inhibited homocysteine-induced cardiomyocyte ferroptosis and upregulated GPX4 expression

**Fig 4:**
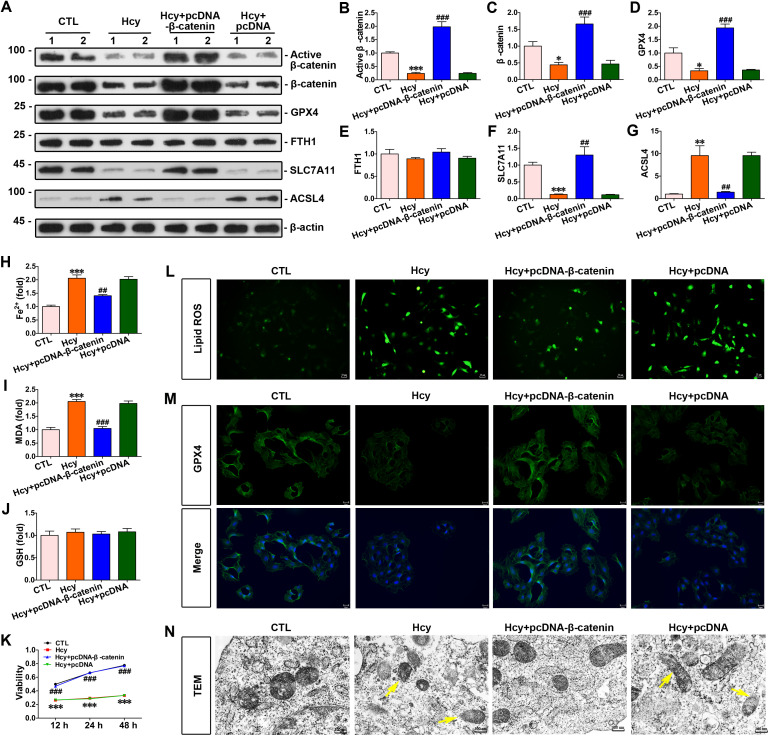
Increased β-catenin expression inhibited homocysteine-induced ferroptosis and upregulated expression of GPX4 in cardiomyocytes. **(A)** Western blots showed protein levels of β-catenin, active β-catenin, GPX4, FTH1, SLC7A11 and ACSL4 in cardiomyocytes of each group as indicated. **(B-G)** Quantitative data on the abundance of specific proteins of Fig 4A were presented in indicated groups. ***P < 0.001, **P < 0.01, *P < 0.05 *vs* the controls; ^###^*P* < 0.001, ^##^*P* < 0.01, ^#^*P* < 0.05 *vs* homocysteine stimulation alone (n = 4). **(H-J)** Quantitative determination of Fe^2+^, MDA and GSH in indicated groups on the colorimetric microplate reader. ***P < 0.001, **P < 0.01, *P < 0.05 *vs* the controls; ^###^*P* < 0.001, ^##^*P* < 0.01, ^#^*P* < 0.05 *vs* homocysteine stimulation alone (n = 4). **(K)** The cell viability was assayed with CCK-8 kit in each group. ***P < 0.001, **P < 0.01, *P < 0.05 *vs* the controls; ^###^*P* < 0.001, ^##^*P* < 0.01, ^#^*P* < 0.05 *vs* homocysteine stimulation alone (n = 4). **(L-M)** Representative micrographs showed immunofluorescent staining for lipid ROS and GPX4 proteins in cardiomyocytes. Scale bar, 25 μm. **(N)** Representative transmission electron microscopy showed mitochondrial damage in cardiomyocytes. The yellow arrows indicated injured mitochondria. Scale bar, 300 nm.

To explore the connection between β-catenin and homocysteine-induced cardiomyocyte ferroptosis, cardiomyocytes were stimulated with homocysteine and transfected with pcDNA3.1-β-catenin. Up-regulated β-catenin expression increased the expression of GPX4; nevertheless, activated β-catenin signaling did not affect FTH1 expression in cardiomyocytes (Figs 4A–4E). SLC7A11 and ACSL4 are two key genes that play critical roles in ferroptosis regulation. SLC7A11 inhibits ferroptosis through its involvement in an antioxidant pathway, while ACSL4 promotes ferroptosis by enhancing lipid peroxidation. Hyperhomocysteinemia decreased the expression level of SLC7A11 while concurrently upregulating ACSL4 expression. In contrast, β-catenin overexpression antagonized the effects of hyperhomocysteinemia by upregulating SLC7A11 expression and downregulating ACSL4 expression (Figs 4F–4G). Simultaneously, increased β-catenin led to a decline in Fe^2+^ and MDA levels in homocysteine-stimulated cardiomyocytes (Figs 4H and 4I). Nevertheless, increased β-catenin expression had no effect on FTH1 and GSH levels in cardiomyocytes (Figs 4E and 4J). Taken together, upregulated β-catenin signaling protected cardiomyocytes against homocysteine-induced ferroptosis. Cell viability was assayed with CCK-8 kit. Homocysteine stimulation reduced cardiomyocyte viability, in contrast, increased β-catenin signaling significantly preserved cell viability against homocysteine at each timepoint (Fig 4K). Lipid ROS levels were quantified with the ﬂuorescent probes C11-BODIPY. As shown in figure 4L, homocysteine significantly increased lipid ROS levels, whereas β-catenin overexpression mitigated the homocysteine-induced increase in lipid ROS. Similarly, immunofluorescence analysis revealed that homocysteine suppressed GPX4 expression compared to the control group. However, β-catenin overexpression restored GPX4 levels in cardiomyocytes (Fig 4M). Moreover, cardiomyocytes were detected on a transmission electron microscope. Electron microscopy showed that homocysteine caused obvious damage to mitochondrion such as membrane disruption, mitochondrial cristae reduction, and mitochondrial shrinkage. Interestingly, β-catenin overexpression preserved mitochondrial integrity against homocysteine-induced damage (Fig 4N).

### GPX4 as a downstream target gene of β-catenin

**Fig 5:**
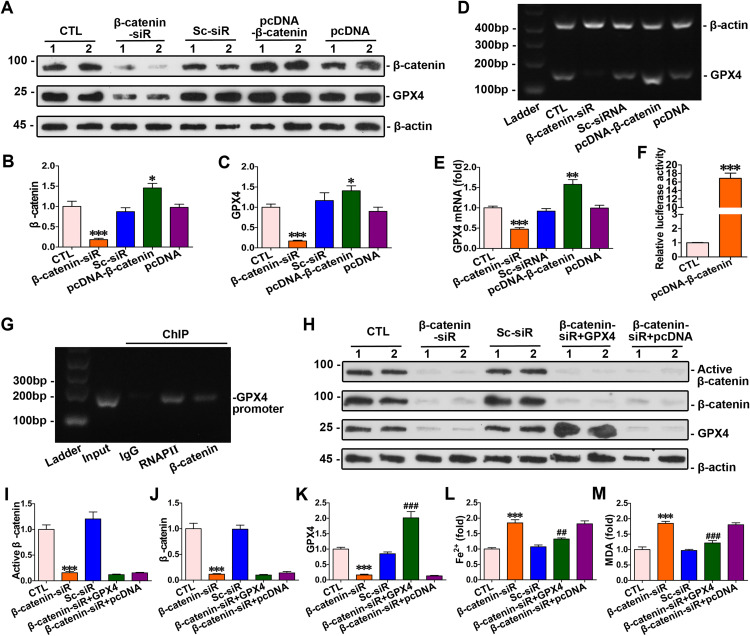
GPX4 acted as a target gene of β-catenin. **(A)** Western blots analysis showed protein levels of β-catenin and GPX4 in cardiomyocytes with overexpression or knockdown of β-catenin. **(B-C)** Quantitative determination of the abundance of specific proteins of Fig 5A were presented in indicated groups. ***P < 0.001, **P < 0.01, *P < 0.05 *vs* the controls (n = 4). **(D)** PCR analysis of GPX4 mRNA levels following β-catenin gene overexpression and silencing. **(E)** Quantitative determination of the abundance of GPX4 mRNA levels in Fig 5D were presented in indicated groups. ***P < 0.001, **P < 0.01, *P < 0.05 *vs* the controls (n = 4). **(F)** Dual luciferase reporter gene assay: both pGL6-TA and pRL-SV40-C were co-transfected into HEK293T cells with pcDNA3.1-β-catenin; both pGL6-TA and pRL-SV40-C were co-transfected into HEK293T cells in the control group. ***P < 0.001, **P < 0.01, *P < 0.05 *vs* the controls (n = 4). **(G)** ChIP assay verified that β-catenin can binded to the specific site on the promoter of GPX4 gene. **(H)** Western blots analysis showed protein levels of active β-catenin, β-catenin and GPX4 in cardiomyocytes of indicated groups. **(I-K)** Quantitative determination of the abundance of specific proteins of Fig 5H were presented in indicated groups. ***P < 0.001, **P < 0.01, *P < 0.05 *vs* the controls; ^###^*P* < 0.001, ^##^*P* < 0.01, ^#^*P* < 0.05 *vs* β-catenin siRNA transfection alone (n = 4). **(L-M)** Quantitative determination of Fe^2+^ and MDA in indicated groups on the colorimetric microplate reader. ***P < 0.001, **P < 0.01, *P < 0.05 *vs* the controls; ^###^*P* < 0.001, ^##^*P* < 0.01, ^#^*P* < 0.05 *vs* β-catenin siRNA transfection alone (n = 4). Sc-siRNA, Scramble-siRNA.

The results above indicated that β-catenin modulated homocysteine-induced ferroptosis through GPX4 in cardiomyocytes. To elucidate the relationship between β-catenin and GPX4, we altered β-catenin expression by transfecting cardiomyocytes with β-catenin siRNA and pcDNA3.1-β-catenin. β-catenin overexpression led to an increase in both protein levels and mRNA of GPX4 in cardiomyocytes. Conversely, Silencing β-catenin decreased GPX4 expression in myocytes (Figs 5A–5E). Intervention experiments on β-catenin expression revealed that GPX4 was a target gene of β-catenin. Moreover, dual luciferase reporter gene assay combining ChIP confirmed the interaction between β-catenin and the GPX4 gene promoter, verifying that GPX4 was a target gene of β-catenin (Figs 5F–5G). To explore the interaction mechanism between β-catenin and GPX4, β-catenin siRNA and GPX4 expression plasmid were co–transfected into cardiomyocytes. The results showed that the knockdown of β – catenin remarkably suppressed the expression level of GPX4 (Figs 5H–5K). Moreover, the knockdown of β – catenin led to a significant increase in the intracellular Fe²⁺ and MDA in cardiomyocytes. Conversely, the overexpression of GPX4 significantly decreased the intracellular levels of Fe²⁺ and MDA in these cells (Figs 5L–5M).

## Discussion

**Fig 6:**
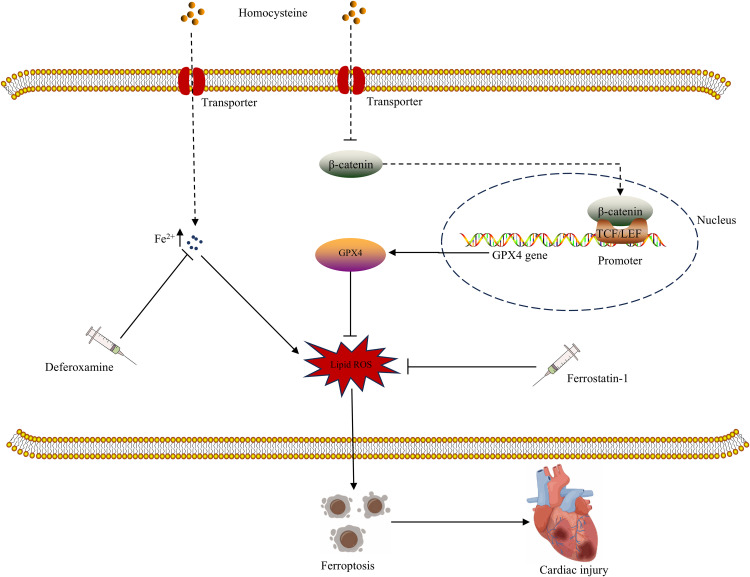
A graphical abstract of the mechanism underlying homocysteine inducing ferroptosis.

Our study shows that homocysteine can induce ferroptosis in cardiomyocytes. The specific mechanism governing homocysteine-induced ferroptosis is that homocysteine downregulates the transcription factor β-catenin, and then downregulates the downstream gene GPX4 (Fig 6). As a crucial inhibitor of ferroptosis, GPX4 deficiency can promote ferroptosis in cardiomyocytes. At present, emerging clinical studies have indicated a connection between hyperhomocysteinemia and multiple cardiovascular diseases [18,19], but the mechanisms by which homocysteine contributes to the pathophysiological process underlying heart disease are rarely explored. In this study, hyperhomocysteinemia was induced in mice by providing drinking water with high levels of homocysteine to investigate the mechanism underlying homocysteine-induced myocardial injury. The results show that ferroptosis is a characteristic pathological feature of myocardial injury induced by homocysteine. Ferroptosis represents a type of regulated cell death dependent on Fe^2+^ [20]. Previous research has indicated a link between ferroptosis and several cardiovascular diseases, including atherosclerosis, heart failure, arrhythmia, myocardial ischemia and perfusion injury, and diabetic cardiomyopathy [21]. Additionally, studies on diseases in other organs have identified a correlation between homocysteine-induced injury and ferroptosis [22]. In this study, homocysteine simultaneously induces the downregulation of both β-catenin and GPX4. As a highly conserved signaling molecule, activated β-catenin signaling promotes cell proliferation and repair in response to stresses such as inflammation and injury [11–13]. Therefore, the downregulation of β-catenin in cardiomyocytes induced by homocysteine may be associated with ferroptosis. Surprisingly, elevated levels of β-catenin attenuate homocysteine-induced ferroptosis and enhance GPX4 expression in cardiomyocytes. GPX4, a pivotal inhibitor of ferroptosis, facilitates the conversion of lipid ROS to deoxidized lipid [23]. The findings of our study indicate that β-catenin regulates GPX4 expression. As expected, ChIP assay results suggest that GPX4 acts as a target gene of β-catenin. Mechanistically, homocysteine lowers GPX4 expression by downregulating β-catenin; as a key inhibitor of ferroptosis, the downregulation of GPX4 subsequently exacerbates ferroptosis. Emerging evidence suggests that β-catenin and GPX4 cooperatively regulate tumor progression and chemotherapy response through ferroptosis modulation. Mechanistically, β-catenin exerts both direct and indirect transcriptional regulation over GPX4 expression, consequently influencing ferroptosis. Notably, Wnt serves as a key upstream activator of β-catenin signaling in tumor cells, playing a pivotal role in this regulatory network [24,25]. Maybe, Wnt is a crucial signaling molecule mediating the action of homocysteine on β-catenin. The present study finds that homocysteine increases the concentration of intracellular Fe^2+^ and initiates ferroptosis, however, increased β-catenin signaling alleviates homocysteine-stimulated ferroptosis and simultaneously decreased the concentration of Fe^2+^. More studies are needed to explore the mechanisms by which homocysteine increases Fe² ⁺ levels and β-catenin signaling reduces Fe² ⁺ accumulation. Anyway, This study provides primary evidence that elevated homocysteine levels directly modulate GPX4 expression in cardiomyocytes through β-catenin-dependent regulation, ultimately triggering ferroptosis. Our study partially elucidates the mechanism underlying homocysteine-induced ferroptosis in cardiomyocytes. Our study confirms the detrimental effects of homocysteine on the heart and elucidates the specific mechanism by which homocysteine initiates ferroptosis in cardiomyocytes. Currently, there is a lack of clinical data on the dysregulation of β-catenin/GPX4 in patients with hyperhomocysteinemia. Based on our research findings, β-catenin or GPX4 may serve as potential biomarkers and therapeutic targets for hyperhomocysteinemia-associated myocardial injury.

## Conclusion

Our study confirms that homocysteine can induce ferroptosis in cardiomyocytes. This process is mediated through the inhibition of β-catenin activation and the subsequent suppression of GPX4, suggesting a distinct pathway by which homocysteine promotes ferroptosis. As a target gene of β-catenin and crucial inhibitor of ferroptosis, GPX4 is inhibited, ultimately triggering ferroptosis. Our study identifies ferroptosis as a crucial mechanism underlying homocysteine-induced cardiac injury and elucidates its specific pathogenic process. Hence, our findings prospectively provide a promising strategy for mitigating cardiac damage in patients with hyperhomocysteinemia.

## Supporting information

S1 Data Original uncropped blot/gel images. (PDF)

S2 Data The data underlying findings. (XLSX)
