# Supplementary figures and images for: Homocysteine induces ferroptosis in cardiomyocytes by disrupting β-catenin/GPX4 pathway
Source: PLoS One. 2025 Aug 6;20(8):e0329792. doi: 10.1371/journal.pone.0329792 (PMC12327623; doi:10.1371/journal.pone.0329792)

Fig 1

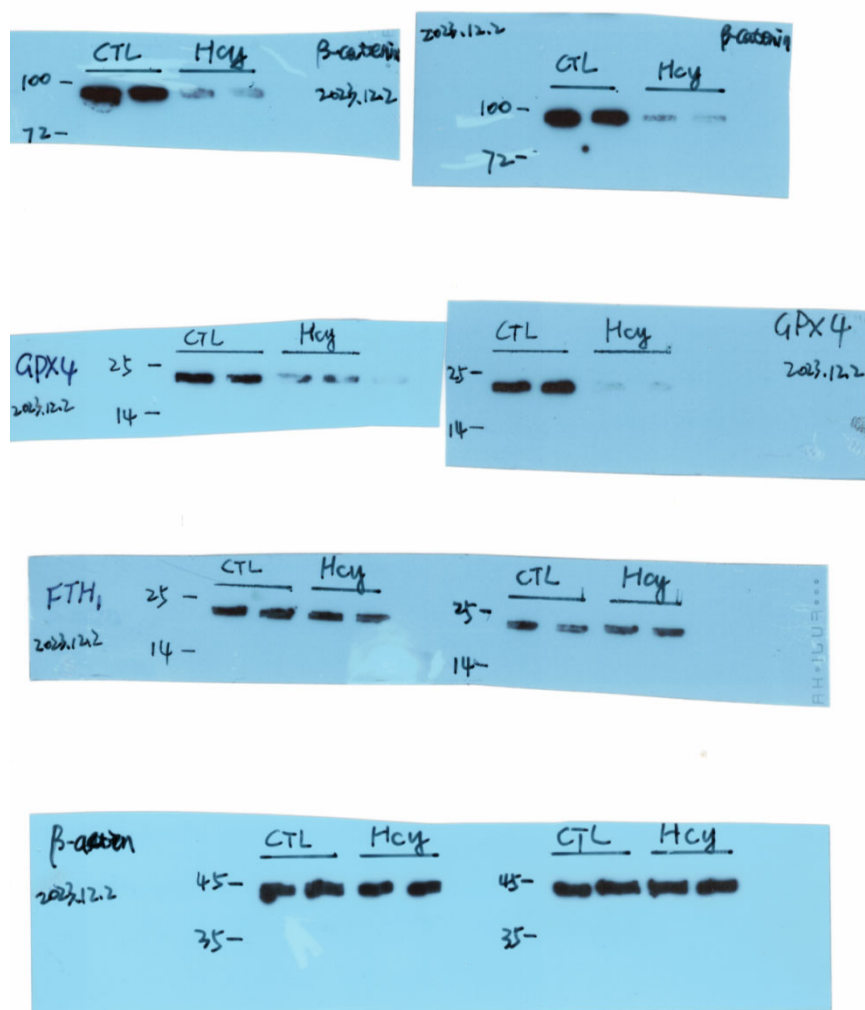

Fig 2

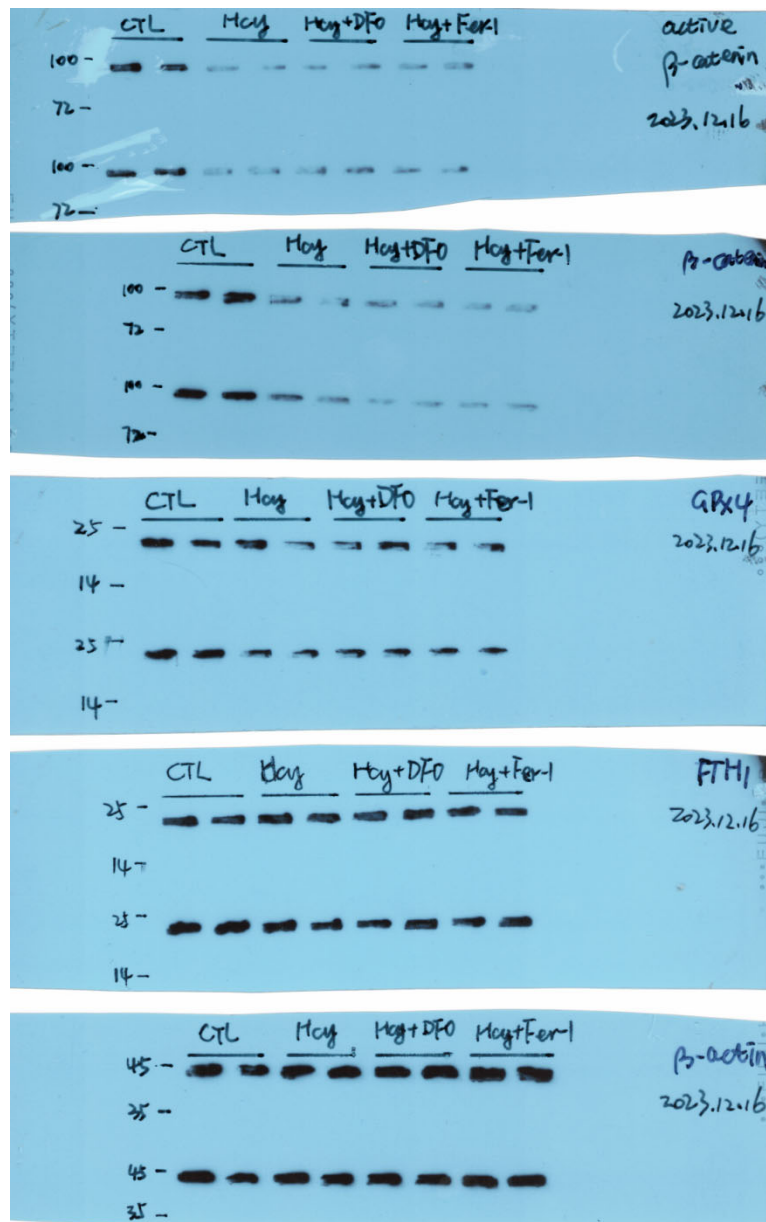

Fig 3

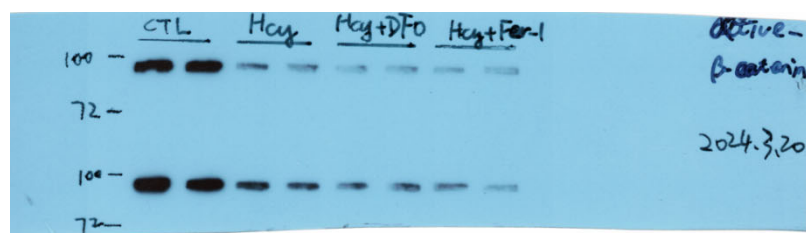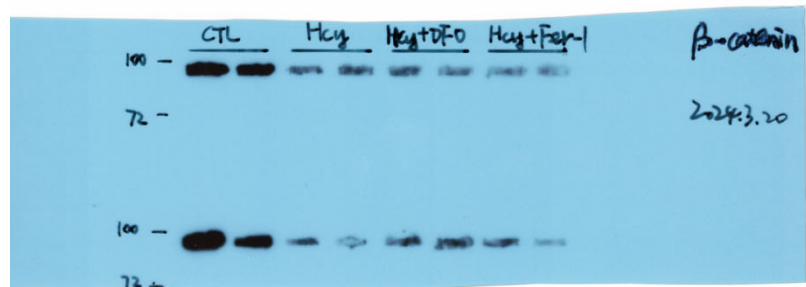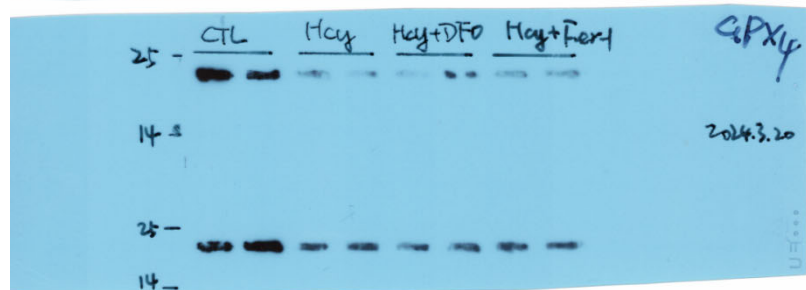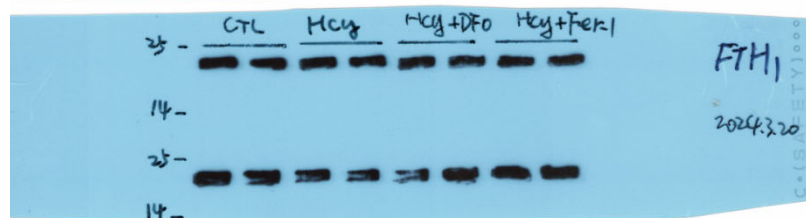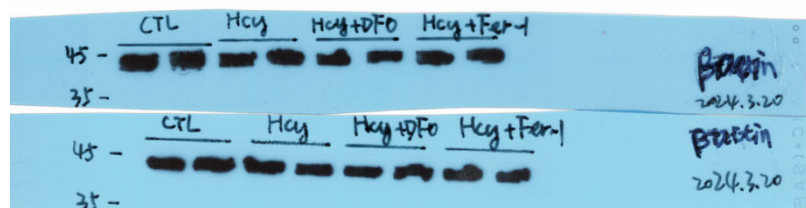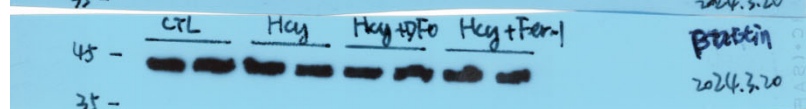

Fig 4

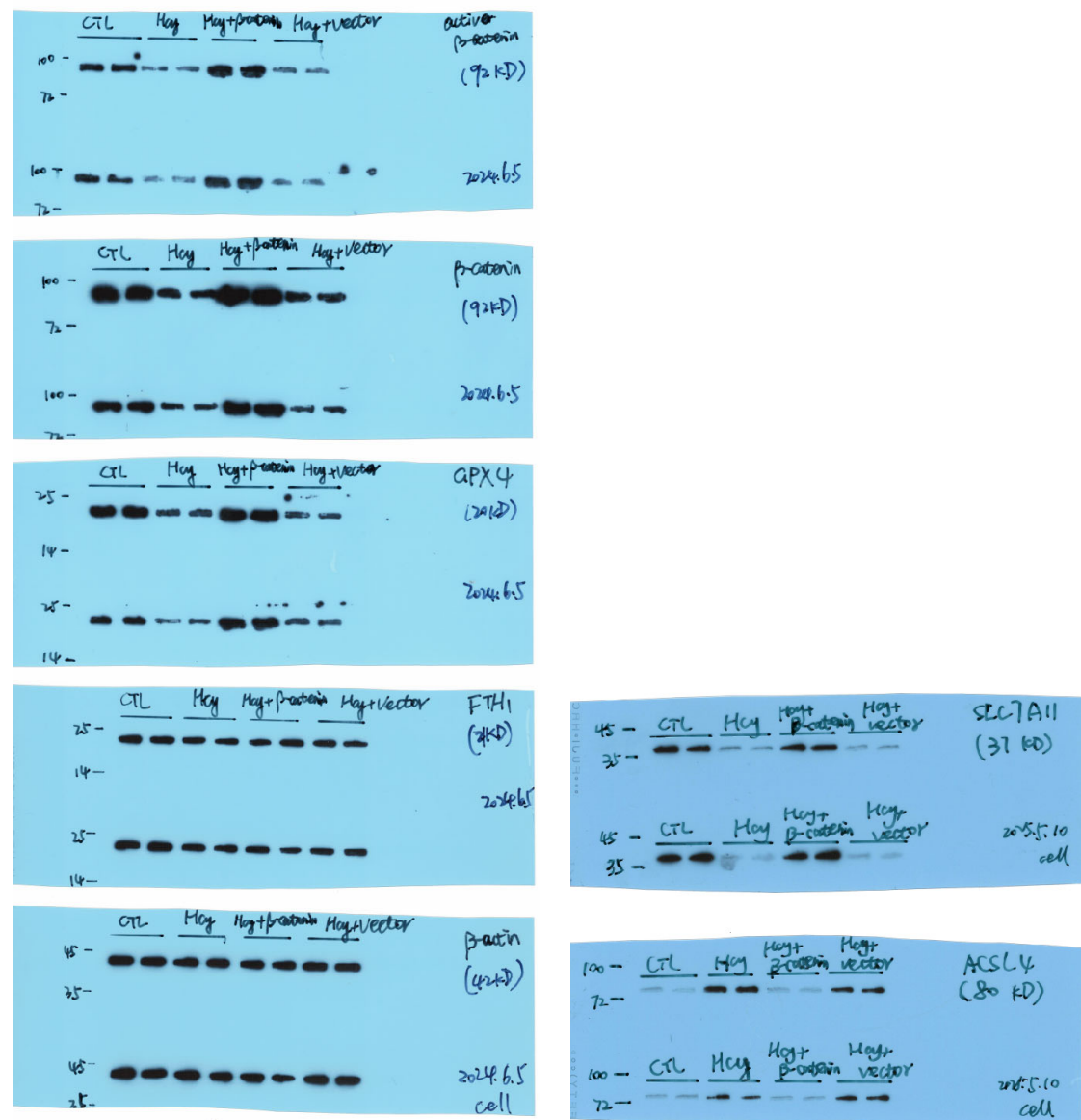

Fig 5A

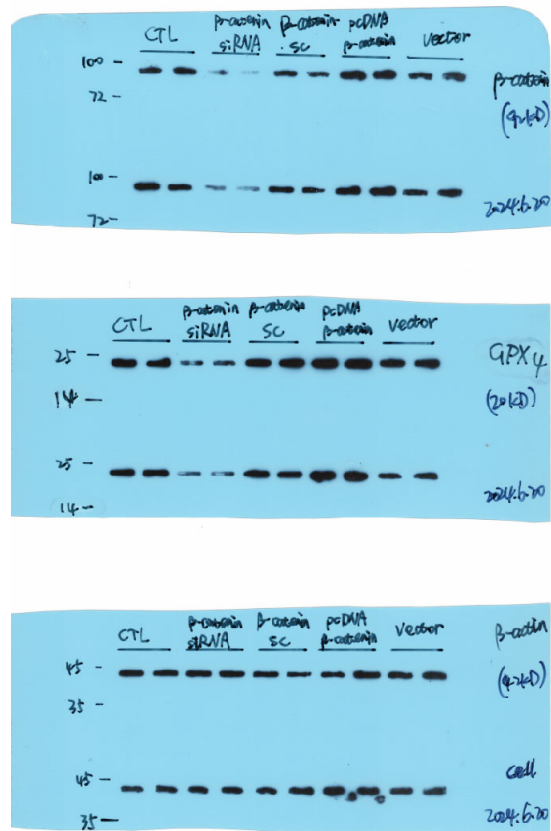

Fig 5H

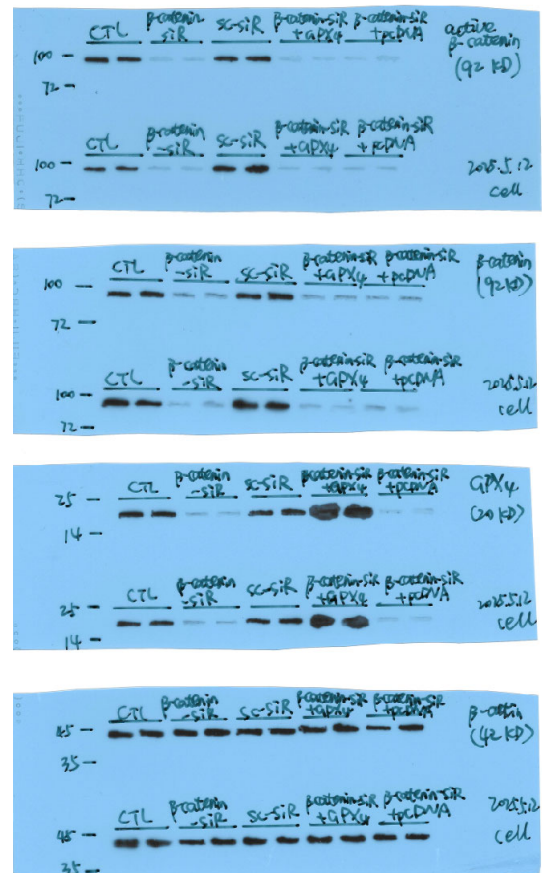

Supplement: S1 Data — (PDF) [file pone.0329792.s001.pdf]
